# The Polycomb BMI1 Protein Is Co-expressed With CD26+ in Leukemic Stem Cells of Chronic Myeloid Leukemia

**DOI:** 10.3389/fonc.2018.00555

**Published:** 2018-12-06

**Authors:** Sara Galimberti, Susanna Grassi, Claudia Baratè, Francesca Guerrini, Elena Ciabatti, Francesca Perutelli, Federica Ricci, Giada Del Genio, Marina Montali, Serena Barachini, Cecilia Giuliani, Maria Immacolata Ferreri, Angelo Valetto, Elisabetta Abruzzese, Chiara Ippolito, Alessandra Iurlo, Monica Bocchia, Anna Sicuranza, Bruno Martino, Lorenzo Iovino, Gabriele Buda, Serena Salehzadeh, Mario Petrini, Antonello Di Paolo, Letizia Mattii

**Affiliations:** ^1^Section of Hematology, Department of Clinical and Experimental Medicine, University of Pisa, Pisa, Italy; ^2^GeNOMEC School of Doctorate, University of Siena, Siena, Italy; ^3^Unità Operativa Cytogenetics, Azienda Ospedaliero-Universitaria Pisana, Pisa, Italy; ^4^Unità Operativa Ematologia, Ospedale S. Eugenio, Rome, Italy; ^5^Section of Histology, Department of Clinical and Experimental Medicine, University of Pisa, Pisa, Italy; ^6^Hematology Division, Foundation IRCCS Ca' Granda Ospedale Maggiore Policlinico, Milan, Italy; ^7^Unità Operativa Ematologia, Università di Siena, Siena, Italy; ^8^Unità Operativa Ematologia, Ospedale Binco, Melacrino, Morelli, Reggio Calabria, Italy; ^9^Section of Pharmacology, Department of Clinical and Experimental Medicine, University of Pisa, Pisa, Italy

**Keywords:** BMI1, polycomb, BCR-ABL1, CML, CD26, leukemic stem cell

## Abstract

The Polycomb gene *BMI1* expression exerts a negative predictive impact on several hematological malignancies, such as acute and chronic myeloid leukemia (CML), myelofibrosis, and follicular lymphoma. As already demonstrated in CML, *BMI1* is responsible for the resistance to the tyrosine kinase inhibitors (TKIs) in a *BCR-ABL1*-independent way. Even if, it is unknown where *BMI1* in CML is expressed (in progenitors or more mature cells). We decided, therefore, to evaluate if and where the BMI1 protein is located, focusing mainly on the CD34+/CD38-/CD26+ CML progenitors. To begin we measured, by flow cytometry, the proportion of CD34+/CD26+ cells in 31 bone marrow samples from 20 CML patients, at diagnosis and during treatment with imatinib. After that the bone marrow blood smears were stained with antibodies anti-CD26, BCR-ABL1, and BMI1. These smears were observed by a confocal laser microscope and a 3D reconstruction was then performed. At diagnosis, CD34+/CD26+ cells median value/μL was 0.48; this number increased from diagnosis to the third month of therapy and then reduced during treatment with imatinib. The number and behavior of the CD26+ progenitors were independent from the *BCR-ABL1* expression, but they summed up what previously observed about the *BMI1* expression modulation. In this work we demonstrate for the first time that in CML the BMI1 protein is co-expressed with BCR-ABL1 only in the cytoplasm of the CD26+ precursors; on the contrary, in other hematological malignancies where BMI1 is commonly expressed (follicular lymphoma, essential thrombocytemia, acute myeloid leukemia), it was not co-localized with CD26 or, obviously, with BCR-ABL1. Once translated into the clinical context, if BMI1 is a marker of stemness, our results would suggest the combination of the BMI1 inhibitors with TKIs as an interesting object of research, and, probably, as a promising way to overcome resistance in CML patients.

## Introduction

Chronic myeloid leukemia (CML) is a hematological malignancy characterized by the translocation between the chromosome 9 and 22, with the consequent generation of the *BCR/ABL1* oncogene, responsible for increased proliferation, reduced apoptosis, and uncontrolled enter into the bloodstream of the immature myeloid cells (1).

After the introduction of the tyrosine kinase inhibitors (TKIs) into the clinical armamentarium, the prognosis of CML has really improved, with a survival target now comparable to that of age-matched healthy subjects (2, 3). Nevertheless, about one third of the patients during the treatment has to change the TKI for occurrence of resistance or intolerance.

There are several mechanisms leading to the resistance, which haven't been well identified yet: in some cases, ABL1 mutations impair the insertion of the TKI into the ATP-binding pocket of the fusion protein (4), but in the majority the activation of alternative pro-proliferating pathways, such as *JAK-STAT, B-catenin/Wnt, Hedgehog*, or the abnormal epigenetic control, can occur (5, 6).

It has been also reported that the hypoxic bone marrow niche protects the leukemic stem cell (LSC) from the TKIs, blocking the BCR-ABL1 protein synthesis (7). Moreover, *BCR-ABL1* fusion gene can still be detected by polymerase chain reaction (PCR) on the genomic DNA in cases not expressing the fusion gene at the mRNA level.

In this way we have the demonstration that LSC can still survive in a quiescent status, even in subjects in deep molecular response (DMR) (8).

Furthermore, the immunological control, including the KIR haplotype (9) and the NK number/activation (10), seems to collaborate to the maintenance of the treatment-free-remission (TFR), which can be reached and maintained by half of patients in persistent DMR (11, 12).

Once translated into the clinical context, these evidences indicate a right way to reach the CML eradication: the addition of TKIs to further drugs able to modify the bone marrow microenvironment or to block the alternative pathways sustaining the LSC survival.

Regarding the LSC identification, in 2014 Hermann and colleagues identified the enzyme dipeptidyl-peptidase-IV (DPPIV/CD26) as a specific marker of the CD34+/CD38- leukemic progenitors, demonstrating in a murine model that these cells were able to summarize the CML onset and that they decreased remarkably during the successful treatment with imatinib (13).

CD26 is able to disrupt the SDF-1-CXCR4-axis, thus detaching the LSCs from the bone marrow niche and increases cell cycling so leading to an increased ability to be targeted by TKIs.

It is very interesting to notice that CD26 antigen is not detectable in normal hematopoietic progenitors or in cells from other hematological malignancies, including acute myeloid leukemia (14).

Our group has also recently measured, by flow cytometry, the CD34+/CD26+ LSCs in bone marrow and peripheral blood from CML patients at diagnosis, during treatment with TKIs and in TFR, confirming that CD26 is a CML-specific marker (15). Interestingly, CD26 was also expressed by a small part of patients in TFR, suggesting that the *BCR-ABL1* transcript measure in the peripheral blood is not always enough to understand the behavior of the LSC in the bone marrow niche, cell that could sustain the loss of DMR or cause the resistance to TKIs.

About the *BCR-ABL1*-independent mechanisms of resistance, we previously focused on the epigenetic control, showing that time to complete cytogenetic response and event-free survival of patients receiving imatinib were negatively conditioned by the expression of some Polycomb genes. In particular, high levels of *BMI1*, an oncogene highly expressed in acute myeloid leukemia and in advanced phases of CML, were connected to a poorer outcome, whereas on the other side a high expression of *CBX6* and *CBX7* played a favorable role (16).

With these premises, we decided to combine the two relevant LSC markers (CD26 and the BMI1) (17), in order to test if they were co-expressed in CML progenitors. Indeed, the possibility of finding a co-expression of BMI1 and CD26 in CML-LSC could have as clinical consequence the possibility of using the *BMI1* inhibitors (today available for the clinical use) for attempting to eliminate LSCs and eventually overcome the resistance to TKIs.

For this purpose, we analyzed, by the confocal microscopy, the expression of CD26, BCR-ABL1 P210, and BMI1 proteins in bone marrow samples from 20 CML patients.

## Patients and Methods

### Patients

Overall, 31 bone marrow samples from 20 patients affected by CML at diagnosis or during the first 12 months of treatment with imatinib were assessed, by flow cytometry, for the presence of CD34+/CD38-/CD26+ cells.

Overall, 21 samples showed CD34+/CD26+ cells in the bone marrow (negative cases were observed after 6 and 9 months of therapy); among them 9 samples with the highest CD26+ values have been then tested for the co-expression of CD26, P210, and BMI1 proteins by the confocal microscopy.

The *BCR-ABL1/ABL1* ratio was measured by quantitative PCR on the concomitantly harvested peripheral blood, according to the standardized operative procedures edited in 2016 by the Italian cooperative group GIMEMA LabNET (www.gimema.it/labnet-cml/).

A minimum of 20,000 ABL1 copies was necessary to consider a sample as “evaluable”; 32,000 ABL1 copies were necessary to define the MR4.5 or 100,000 ABL1 copies for MR5, according to the ELN guidelines (18).

The enrolled patients were consecutively observed at the Hematology units of Pisa, Milano, Siena, Reggio Calabria, and Roma (Italy) and enrolled only on the basis of their acceptance to take part to the study. Each of them signed an informed consent to donate the residues of the material harvested for the routine diagnostic tests for further no-profit scientific purposes. This informed consent was previously approved by the respective Ethical Committees.

The clinical characteristics of patients are reported in Table 1.

**Table 1:** Clinical characteristics on the 20 patients enrolled into the study.

**Clinical feature**	***n* (%)**
Patients	20
Age (median/range)	64 (56–79)
**SEX**	
M	11 (55)
F	9 (45)
**SOKAL RISK SCORE**	
Low	7 (35)
Intermediate	9 (45)
High	4 (20)
**TKI**	
Imatinib	20 (100%)

### Flow-Cytometry Assay

For each patient 2 mL of bone marrow samples collected in EDTA tubes were analyzed. CD26 expression was assessed by multiparametric flow cytometry, analyzing the CD45^+^/CD34^+^/CD38^−^ population using a four-color protocol, as previously reported by our group (15). Briefly, 2.0 x 10^6^ leucocytes were incubated with BD Pharmigen CD45-V500 (clone 2D1), CD34-FITC (clone 581), CD38-APC (clone HIT2), CD26-PE (clone M-A261), and proper negative controls. After washing, acquisition and analysis of at least 1.0 × 10^6^ of CD45^+^ cells were performed by using a MACSQuant Analyzer (Miltenyi, Gemany), equipped with 3 lasers and 8 fluorescent channels available. The absolute number of CD26+ cells was calculated by multiplying the number of white cells/μL automatically counted for the proportion of CD34^+^/CD38^−^/CD26^+^ on CD45^+^ cells using the MACSQuantify Software (Miltenyi).

### Immunofluorescence Assays

#### Triple-Immunofluorescence

From each sample, 4 smears were prepared using the bone marrow, then fixed in cold acetone/methanol (1:1 v/v) for 10 min at −20°C, washed in phosphate buffered saline (PBS 1x), and treated for 10 min with 0.2% triton-X100/PBS. After 1 h in blocking solution (BS, 0.1% Tween, 0.25% BSA in PBS), slides were incubated overnight at 4 °C with the following primary antibodies diluted in BS at optimal working dilutions, determined empirically by means of serial dilutions: MαBCR/ABL1 (1:300, Thermo Fisher, Rockford, IL, USA), RαBMI-1 (1:100, Thermo Fisher), and GαCD26 (1:100, R&D system, Minneapolis, MN, USA). Slides were then washed three times in BS and incubated in the dark with fluorescent conjugate secondary antibodies diluted 1:250 in BS; a first incubation of 90 min with DαR and DαG antibodies (Alexa Fluor® 568 and Alexa Fluor® 647, Life Technologies, Monza, Italy) was performed.

After three washes in BS, a second incubation for 90 min with GαM antibody (Alexa Fluor® 488 Life Technologies Italia) was made. The slides were sequentially washed in BS, PBS 1x, and finally mounted in mounting medium with DAPI (Sigma-Aldrich, St. Louis, MO, USA). All steps were performed at room temperature unless otherwise specified. The samples were then observed at 20x, 43x, or 63x magnification with a confocal laser scanning microscope (TC SSP8 Leica Microsystems, Mannheim, Germany) using 488-nm, 561-nm, and 642-nm excitation wavelength lasers.

Negative controls for secondary antibodies were performed omitting primary antibodies. The analysis of immunofluorescence pattern was performed in at least three fields per smear for each sample. Confocal multiple optical sections (z-step = 0.35 μm) were used to obtain the maximum intensity projections and the iso-surface representation.

In addition, as disease-negative controls, 2 mL of bone marrow from one patient with essential thrombocytemia, one with hypereosinophilia, two with MGUS, one with multiple myeloma and one with non Hodgkin's follicular lymphoma were also assessed.

### Statistical Analysis

All statistical analyses were carried out using the SPSS software, version 22.0 (SPSS, IBM, Bologna, Italy). In all computations, the level of significance was set at *p* < 0.05.

To analyze differences in the levels of each single variable, we employed the Mann–Whitney *U*- and the Wilcoxon-tests. The Kendall one-tail correlation test was used to detect relationships between two measurable variables and the *t*-test for comparison of mean values. For comparison of the median values, the median test was adopted. The linear regression was adopted for comparing CD34+ and CD26+ values.

## Results

### Patients' Characteristics and CD34+/CD26+ Myeloid Progenitors

Before evaluating the expression of the BMI1 protein in CD34+/CD26+ progenitors (whose CML identity was confirmed by the BCR-ABL1 protein expression in order to avoid the mature CD26+ lymphocytes), we identified among the 31 samples from the 20 patients enrolled into the study those with measurable levels of CD34+/CD26+ precursors.

In 21 samples we were able to detect and measure the CD34+/CD26+ cells; all samples at diagnosis and 11 during the follow-up were positive, whereas 10 cases harvested at 6 and 9 months resulted negative. Among these samples CD26-positive, we chose for the microscopic analysis 9 samples with the highest CD26/uL levels.

The median age of the enrolled patients (11 men and 9 women) was 64 years (range, 56–79). At diagnosis, all of them were in chronic phase; the median number of blasts in the bone marrow was 4%; the Sokal risk score was high in 4, intermediate in 9, and low in 7 cases. All patients received imatinib as first line of therapy and during the study. Clinical characteristics of patients are detailed in Table 1.

All patients enrolled were tested at diagnosis; during the follow-up, 13 bone marrow samples were harvested by 12 months of therapy.

Firstly, we used flow cytometry to identify and measure the CD34+/CD38− cells on the samples at diagnosis: their median value was 130/uL (range, 24–810). After 3 months of imatinib, the median value of the CD34+/CD38− progenitors was 14.4 (range, 0.21–575); after 6 months, 78.1 (range, 15.7–1250); after 9 months, 41.1 (range, 4.4–397); after 12 months, 73.8 (range, 0.1–503). The values at 15 months were not computed, because of the small number (2 patients were evaluated at this time-point).

After the median test, no statistically significant differences were noticed for the value of CD34+ cells/uL among the different considered time-points.

Then, we measured the CD34+/CD38-/CD26+ fraction: at diagnosis, its median value was 0.33/uL (range, 0.9–12.05). After 3 months of imatinib, the median value increased up to 2.01 (range, 1.56–13.9); after 6 months, to 2.87 (range, 0.48–5.23); after 9 months, the median number of CD26+ cells decreased to 0.11 (range, 0–2.52); after 12 months, it was 0.23 (range, 0–0.26).

The difference measured between the month +6 and the month +12 resulted statistically significant (2.87/uL after 6 months of imatinib vs. 0.23/uL; after 12 months; *p* = 0.04).

**Figure 1:**
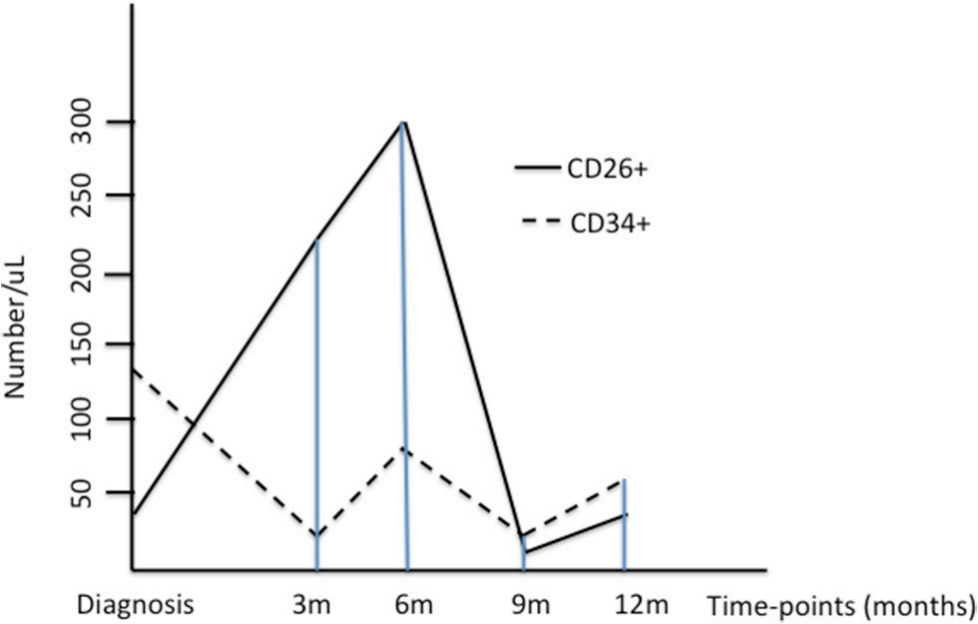
The Figure represents the median values of CD34+/CD38- and CD34+/CD26+ progenitors at diagnosis and during treatment with imatinib; for a better understanding, the values of CD26+ cells have been multiplied by ten.

The behavior of CD34+/CD38- and CD34+/CD38-/CD26+ progenitors is represented in the Figure 1.

When linear regression was performed between CD34+ and CD26+ values, as expected, a significant correlation was found (*p* = 0.008).

**Figure 2:**
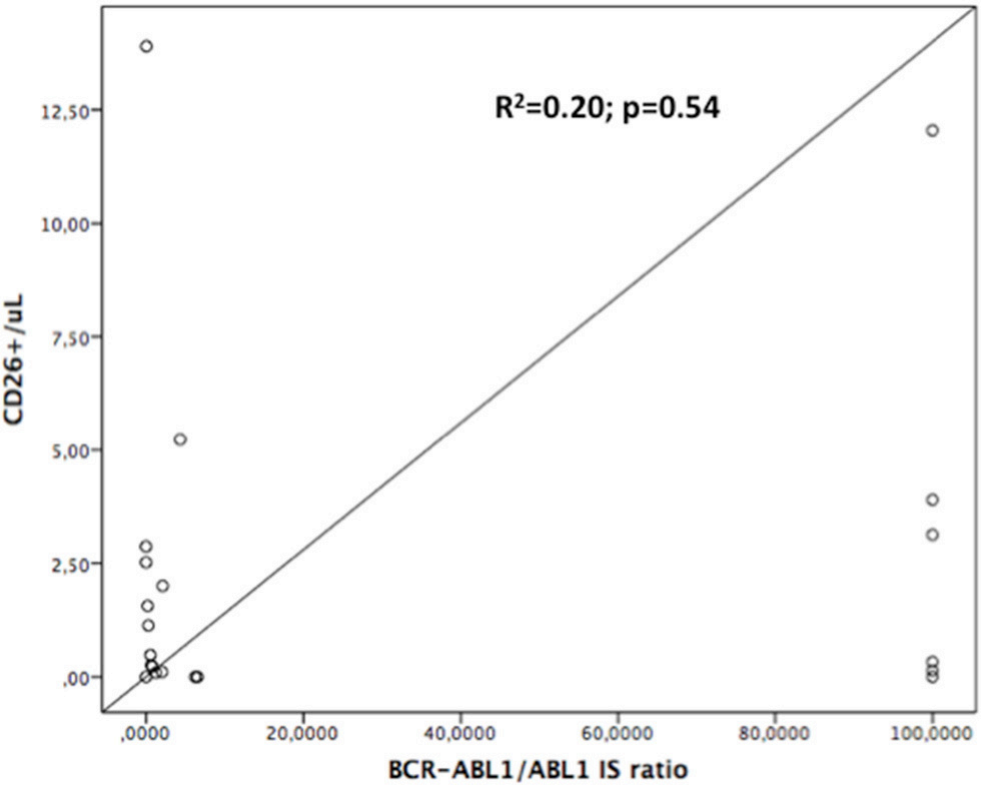
Linear regression curve: CD34+/CD26+ number is not significantly correlated with *BCR-ABL1/ABL1* expression, either at diagnosis or during follow-up. *R*^2^ = 0.20; *p* = 0.54.

On the contrary, when we assessed if the number of the CD26+ progenitors in bone marrow was correlated with the *BCR-ABL1* mRNA expression assessed in the peripheral blood, no significant relationship has been found (*R* = 1.42; *R*^2^ = 0.20; *p* = 0.54; see Figure 2).

As above reported, at diagnosis (where *BCR-ABL1/ABL1* ratio has been conventionally considered as 100%) the median value of CD26+ progenitors was 0.33/uL. It is interesting to note that in 3 cases the CD26+ value at diagnosis was inferior in respect of the median one; in 2 of these 3 cases, also the values of CD34+/CD38- cells were very low (65 and 24/uL vs. the median = 130/uL). Interestingly, these two cases revealed as optimal responders, because both reached either the early molecular response or the MR4 by the 12th month.

In our series, only one patient developed a blastic phase, concomitantly to the detection of T315I compound mutation. Nevertheless, at the time of this study, he was still in response, showing a low number of CD26+ cells.

**Figure 3:**
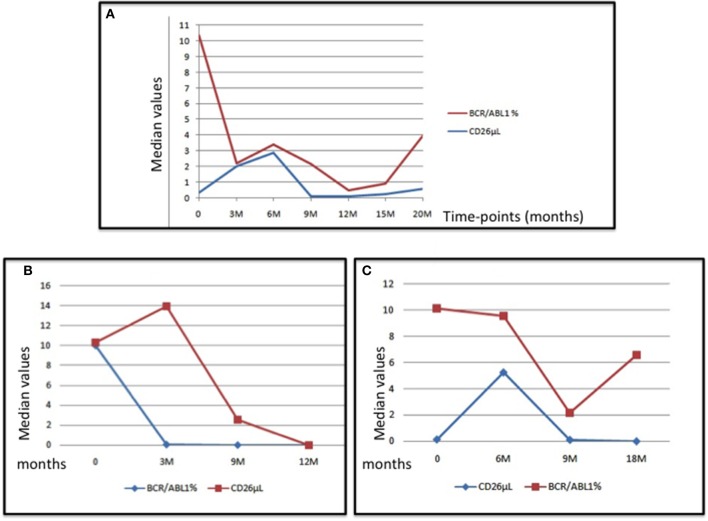
The Figure represents the median values of CD26+ cells and of the *BCR-ABL1/ABL1* transcript at diagnosis and during treatment with imatinib in the whole series (box **A**), in an “optimal” case (box **B**), and in a “warning” case (box **C**).

The behavior of the median number of CD26+ progenitors from the bone marrow and of the *BCR-ABL1/ABL1* ratio measured in the peripheral blood of the whole series (box A), of a “optimal” case (box B) and of a “warning” subject (box C) are represented in the Figure 3.

Finally, the number of CD26+ cells measured at the different time-points was not associated to the clinical characteristics of our patients (sex, age, or Sokal risk).

## Confocal Microscopy

For the microscopy analysis, we selected 9 samples showing the highest number of CD34+/CD26+ precursors (median value, 3.13/uL, range 1.56–13.09).

**Figure 4:**
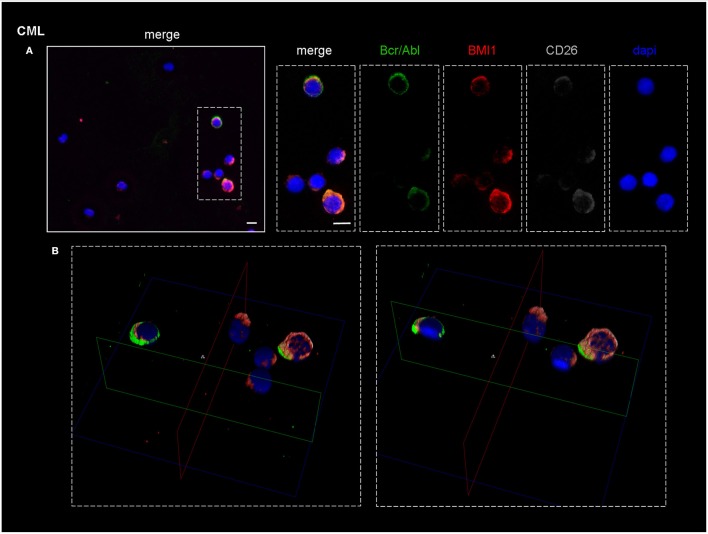
Confocal laser scanning microscopy: representative images of a bone marrow smear from one of the 9 CML patients enrolled into the study. **(A)** Bidimensional images of the maximum intensity projection. Scale bars 10 μm. From the right to the left: in blue, the nuclei; in gray, the CD26+ cells; in green, cells expressing BCR-ABL1. The merged imagines show that CD26+ cells co-express both BMI1 and BCR-ABL1. **(B)** 3D-reconstruction of the merged imagines: BMI1 and BCR-ABL1 are co-expressed in the cytoplasm of the CD26+ cells.

As shown in Figure 4A, which represents one of the CML cases, the BMI1 protein (in red) was co-expressed with CD26+ (in gray) and with the P210 (in green).

Noteworthy, the blue nuclear staining by DAPI identified in the bone marrow smear the presence of some CD26- cells that resulted both P210- and BMI1-negative.

In the B box of the same figure, the tridimensional confocal microscope analysis showed that the 3 target proteins were co-localized in the cytoplasm of the CD26+ LSC (Figure 4B).

This finding characterized all the 9 tested samples.

**Figure 5:**
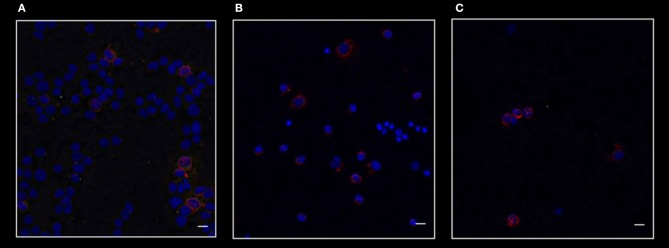
Confocal laser scanning microscopy: representative images of bone marrow smears from patients with acute myeloid leukemia **(A)**, essential thrombocytemia **(B)**, and follicular lymphoma **(C)**. Scale bars 10 μm. Reactions for BMI1 (red), BCR-ABL1 (green), and CD26 (gray). It is clear that in these malignancies only BMI1 is expressed. As expected, these diseases are all CD26- and BCR-ABL1-negative.

Figure 5 shows the merged staining for CD26, P210, and BMI1 in a patient affected by acute myeloid leukemia (A), in one subject with essential thrombocytemia (B), and in one case affected by follicular lymphoma (C). As clearly shown, in these Philadelphia-negative cases BMI1 antigen was present, but only in a small percentage of CD26- cells.

**Figure 6:**
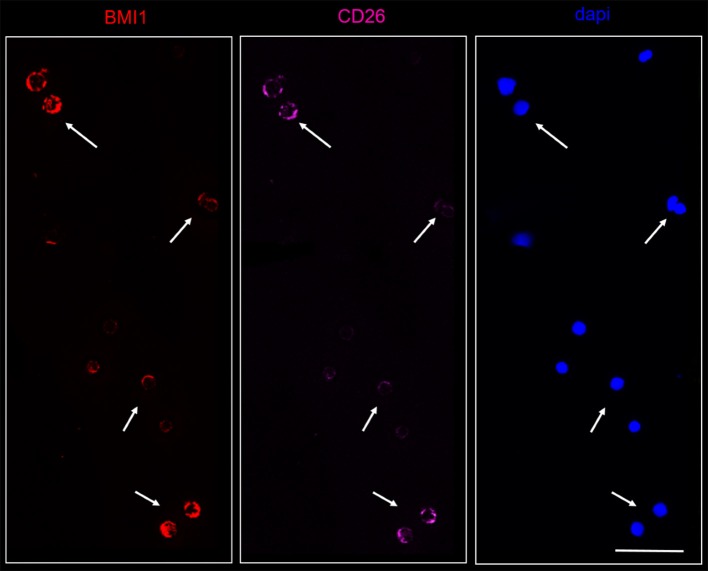
Confocal laser scanning microscopy: representative images of a bone marrow smear from one of the 9 CML patients enrolled into the study with a high representation of CD26+ cells. Bidimensional images of the maximum intensity projection. Scale bars 50 μm. From the right to the left: in blue, the nuclei; in pink, the CD26+ cells; in red, the BMI1+ cells. Cluster of cells marked by the arrows co-expressed CD26 and BMI1 antigens.

On the contrary, BMI1 was co-localized in CD26^+^ cells from all CML samples, as represented in the Figure 6.

This is in accordance to data already reported in literature, where BMI1 positivity has been described in AML blasts, in Ph'-negative chronic myeloproliferative neoplasias, and in follicular lymphoma.

Therefore, our work clearly demonstrates that BMI1 protein can be present in several hematological malignancies, but that only in CD26+ cells (thus CML LSCs) it is co-expressed with the BCR-ABL1 fusion protein.

## Discussion

The Polycomb genes have been reported to play a relevant pathogenetic and predictive role across multiple cancers, including the hematological ones. In acute myeloid leukemia, *BMI1* is over-expressed, while other genes from the same family, such as *CBX6* and *CBX7*, are down-regulated (19). In myelodysplastic syndromes and in clonal hematopoiesis (CHIP), *EZH2* and *TET2* have been reported to be associated with higher rate of cardio-vascular events (20), and *TET2* mutations (at low allelic burden) seem to positively predict the response to azacitidine (21).

In Ph'-negative chronic myeloid neoplasms, *BMI1* seems to be correlated with a higher degree of bone marrow fibrosis (22). Finally, in follicular lymphoma, *BMI1* represents a significant poor factor in terms of overall survival (23). Therefore, in hematology, *BMI1* acts as a “bad” gene, and it could represent an interesting and promising target for new therapies.

In other neoplasias, such as the breast cancer, the *BMI1* knock-down is able to increase the expression of several tumor-suppressive miRNAs (miR-200a, miR-200b, miR-15a, miR-429, miR-203), with the consequent reduced expression of N-Cadherin, Vimentin, and β-Catenin, thus resulting in a decreased invasion, migration and proliferation of the neoplastic cells (24).

In an *in vitro* leukemia model, two *BMI1* inhibitors, PTC-209 and PRT-4165, down-regulating the expression of *NOTCH1, HES1*, and c-*MYC*, were able to block the leukemic cells growth, suggesting that the *BMI1* inhibitors can be good candidates against leukemia (25). Finally, in multiple myeloma, PTC-209 showed a synergistic activity when combined with other epigenetic inhibitors (such as those targeting *EZH2* and *BET*) (26).

With these premises, we supposed that the epigenetic control exerted by the Polycomb family genes could be at least one of the causes of the resistance to TKIs in CML: as we have already demonstrated in our previous work *BMI1* could represent a new valid, predictive marker of response to imatinib, independently from the *BCR-ABL1* behavior or ABL1 mutations (16). In particular, the *BMI1* expression initially increased notwithstanding the reduction of the *BCR-ABL1* transcript, probably because of the precocious elimination of the more sensitive leukemic cells (that could be those carrying lowest *BMI1* levels). After the third month of treatment, the values of *BMI1* expression well correlated with the molecular response, and high BMI1 levels did negatively impact on the event-free-survival.

Nevertheless, our pivotal work did not answer the question about which kind of cells in CML would express *BMI1*; with the purpose to solve this clue, we designed the present study, whose aim was to find if the CD26+ cells, in the meanwhile identified as the true LSC, were the “reservoir” of this protein.

Also if conducted on a small series of patients, we showed that in CML the BMI1 protein was co-expressed with the P210 only in the cytoplasm of the CD26+ cells.

On the other hand, as expected, BMI1 was also detected in other myeloid and lymphoid tumors (essential thrombocytemia, acute myeloid leukemia, follicular lymphoma), but in all these cases it was never co-expressed with CD26 nor, obviously, with P210.

This could suggest that in CML BMI1 could be considered, in addition to the CD26, as a potential marker of stemness.

Unfortunately, we did not perform a quantitative estimation of the BMI1 protein (the confocal microscopy is not a quantitative technique), but it is very intriguing to observe that the CD34+/CD26+ progenitors number increased from diagnosis to the sixth month of treatment, while the overall CD34+ elements and the *BCR-ABL1* transcript decreased, analogously to that we observed about the *BMI1* expression.

This could be probably explained by the more rapid and effective action exerted by imatinib on the more mature cells in comparison with the CD26+ progenitors.

Moreover, in our study we did not find any significant correlation between CD34+/CD26+ cells and *BCR-ABL1* mRNA expression; this observation could confirm that coming from another work published by our group, where CD26+ cells have been detected even in subjects in DMR and during TFR, independently from the TKI used (15).

From the technical point of view, it is well known that at diagnosis the *BCR-ABL1/ABL1* ratio is underestimated for the higher amount of *ABL1* gene, either “normal” or “pathological,” and that *GUS* could be a better reference gene (27), but this technique has not been standardized yet in the Italian network, and thus we considered for all patients at diagnosis a *BCR-ABL1/ABL1* ratio of 100% IS. Nevertheless, also when we focused the statistical analysis only on the follow-up samples, no significant relationship between the number of CD34+/CD26+ cells in the bone marrow and the *BCR-ABL1* expression in the peripheral blood was found.

This observation, even if as an indirect proof and performed only in a small series of cases, could prompt us to hypothesize that (1) *BMI1* is specifically expressed by the CML CD26+ LSC, and (2) because of its *BCR-ABL1*-independent behavior, *BMI1* could be considered as one of the mechanisms of resistance to TKIs, that would make this gene an interesting therapeutic target for new drugs in the CML landscape.

At last the recent availability of the PTC-596, a *BMI1* inhibitor is now being tried against the ovarian cancer (ClinicalTrials.gov Identifier: NCT03206645) and the possibility to realize further *in vitro* and *in vivo* models with cells where *BMI1* could be silenced (for example by the CRISPR approach) probably will give us further demonstration about of a possible translation of our pivotal findings in the clinical scenario of the CML.

Obviously, additional *ex vivo* experiments conducted on larger series of CML patients and with the second- and third-generation TKIs are required to evaluate the potential usefulness of combining the *BMI1* inhibitors with TKIs in resistant cases.

## Ethics Statement

This study was carried out in accordance with the recommendations of Good Clinical Practice. All subjects gave written informed consent to leave leftover from the routine diagnostic samples for further no-profit studies, in accordance with the Declaration of Helsinki.

## Author Contributions

SaG, SuG, MP, LM, AD designed the study, performed statistical analyses, and wrote the manuscript. FG, EC, FP, GD, MIF, AV, CG, CI, LM, and SuG performed the confocal microscope experiments; FR, EA, AI, MB, AS, BM, LI, GB, and CB treated patients and collected data; MM, SS, and SB performed the flow cytometry tests.

### Conflict of Interest Statement

The authors declare that the research was conducted in the absence of any commercial or financial relationships that could be construed as a potential conflict of interest.
